# Effects of supplementation with nondigestible carbohydrates on fecal calprotectin and on epigenetic regulation of *SFRP1* expression in the large-bowel mucosa of healthy individuals^1^,^2^

**DOI:** 10.3945/ajcn.116.135657

**Published:** 2017-01-11

**Authors:** Fiona C Malcomson, Naomi D Willis, Iain McCallum, Long Xie, Idoia Ibero-Baraibar, Wing C Leung, Seamus Kelly, D Michael Bradburn, Nigel J Belshaw, Ian T Johnson, John C Mathers

**Affiliations:** 3 Human Nutrition Research Centre, Institute of Cellular Medicine, Newcastle University, Campus for Ageing and Vitality, Newcastle upon Tyne, United Kingdom;; 4 Institute of Food Research, Norwich Research Park, Norwich, Norfolk, United Kingdom;; 5 Northumbria Healthcare National Health Service Foundation Trust, North Shields, United Kingdom; and; 6 Northumbria Healthcare National Health Service Foundation Trust, Ashington, United Kingdom

**Keywords:** nondigestible carbohydrates, colorectal cancer, calprotectin, Wnt signaling, epigenetics

## Abstract

**Background:** Hyperactive Wnt signaling is frequently observed in colorectal cancer. Higher intakes of dietary fiber [nondigestible carbohydrates (NDCs)] and the fermentation product butyrate are protective against colorectal cancer and may exert their preventative effects via modulation of the Wnt pathway.

**Objectives:** We investigated the effects of supplementing healthy individuals with 2 NDCs [resistant starch (RS) and polydextrose] on fecal calprotectin concentrations and Wnt pathway–related gene expression. In addition, we determined whether effects on secreted frizzled-related protein 1 (*SFRP1*) expression are mediated via the epigenetic mechanisms DNA methylation and microRNA expression.

**Design:** In a randomized, double-blind, placebo-controlled trial (the Dietary Intervention, Stem cells and Colorectal Cancer (DISC) Study), 75 healthy participants were supplemented with RS and/or polydextrose or placebo for 50 d in a 2 × 2 factorial design. Pre- and postintervention stool samples and rectal mucosal biopsies were collected and used to quantify calprotectin and expression of 12 Wnt-related genes, respectively. The expression of 10 microRNAs predicted to target *SFRP1* was also quantified by quantitative reverse transcriptase-polymerase chain reaction, and DNA methylation was quantified at 7 CpG sites within the *SFRP1* promoter region by pyrosequencing.

**Results:** NDC supplementation did not affect fecal calprotectin concentration. *SFRP1* mRNA expression was reduced by both RS (*P* = 0.005) and polydextrose (*P* = 0.053). RS and polydextrose did not affect *SFRP1* methylation or alter the expression of 10 microRNAs predicted to target *SFRP1.* There were no significant interactions between RS and polydextrose.

**Conclusions:** RS and polydextrose supplementation did not affect fecal calprotectin concentrations. Downregulation of *SFRP1* with RS and polydextrose could result in increased Wnt pathway activity. However, effects on Wnt pathway activity and downstream functional effects in the healthy large-bowel mucosa remain to be investigated. The DISC Study was registered at clinicaltrials.gov as NCT01214681.

## INTRODUCTION

A large proportion of colorectal cancer (CRC)^7^ cases are linked to lifestyle factors (1), and it has been estimated that 70% of cases could be prevented by adopting a healthier diet (2). Convincing evidence exists for reduced CRC risk with higher intakes of dietary fiber (3, 4), including nondigestible carbohydrates (NDCs) such as resistant starch (RS). NDCs are fermented by colonic bacteria to produce the SCFAs acetate, butyrate, and propionate. Butyrate is the primary energy source for colonocytes and is key to large-bowel health through regulation of processes, including cell proliferation. Butyrate also possesses anticancer properties through its anti-inflammatory and antiproliferative effects (5). These effects may be mediated by regulation of gene expression through epigenetic mechanisms, primarily histone modifications (6). More recently, studies have shown that butyrate modulates Wnt signaling, a pathway frequently hyperactivated in CRC, providing an additional mechanism through which butyrate may protect against CRC (7).

The canonical Wnt pathway is aberrantly active in ∼90% of all CRCs (8). Wnt signaling is negatively regulated by inhibitors belonging to the secreted frizzled-related protein (SFRP) family that prevent Wnt ligands binding and activating the pathway (9). SFRP1 is a 35.4-kDa protein (10) that has been proposed to be a tumor suppressor because of its role in antagonizing Wnt signaling. Epigenetic silencing of *SFRP1,* as a consequence of promoter hypermethylation, results in increased Wnt pathway activity and is an early event in colorectal tumorigenesis (11, 12). To our knowledge, there are no published studies of the effects of NDCs on *SFRP1* expression or methylation in the large bowel or in CRC cells. However, Shin et al. (13) have investigated the effects of butyrate in human gastric cancer cells, where aberrant Wnt pathway activity also occurs, and found that treatment with butyrate restored *SFRP1* expression, which correlated with promoter demethylation (13).

Altered microRNA expression is another epigenetic mechanism leading to abnormal gene expression and that contributes to colorectal carcinogenesis. MicroRNAs from the oncogenic *miR-17–92* cluster are frequently overexpressed in colorectal, urine, stool, and plasma samples of CRC patients (14–16). In vitro butyrate downregulated expression of members of the *miR-17–92* cluster in HCT-116 and HT29 CRC cells (17). In a randomized crossover trial, a high–red meat diet (associated with greater CRC risk) upregulated microRNAs from the miR-17–92 cluster, and these were restored to baseline levels with the consumption of butyrylated RS (18). These findings suggest that butyrate and NDCs may protect against CRC through effects on microRNAs that are abnormally expressed during colorectal carcinogenesis. A limited number of studies have investigated the regulation of *SFRP1* by microRNAs. In the colon, Fu and colleagues (19) observed an inverse correlation between 26 CRC-specific microRNAs and *SFRP1* mRNA expression.

The aim of this study was to investigate the effects of 2 NDCs, RS and polydextrose, on inflammation (the primary outcome was fecal calprotectin concentration) and on Wnt signaling in the large-bowel mucosa of healthy individuals [the DISC (Dietary Intervention, Stem cells and Colorectal Cancer) Study]. In addition, we investigated the effects of the dietary interventions on *SFRP1* mRNA expression and whether these effects were mediated via altered epigenetic regulation of *SFRP1* by DNA methylation and microRNA expression.

## METHODS

### Study participants

Participants in the DISC Study were recruited from gastroenterology outpatient departments at North Tyneside General Hospital, North Shields, United Kingdom, and Wansbeck General Hospital, Ashington, United Kingdom, between May 2010 and July 2011. All individuals provided informed consent. The exclusion criteria included ages <16 or >85 y; being a prisoner at the time of endoscopy; being pregnant or planning to become pregnant; having a diagnosis of diabetes mellitus, familial adenomatous polyposis syndrome, Lynch Syndrome, or a known colorectal tumor or prior CRC; having had prior colorectal resection; having active colonic inflammation at endoscopy, iatrogenic perforation at endoscopy, incomplete left-sided examination, colorectal carcinoma discovered at endoscopy, or CRC on histology; having received chemotherapy in the last 6 mo; or receiving nonsteroidal anti-inflammatories, anticoagulants, or immunosuppressive medication.

Ethical approval for this project was provided by the Newcastle and North Tyneside Research Ethics Committee from which a favorable ethical opinion was received on 10 December 2009 (REC no. 09/H0907/77). Caldicott approval for the storage of data was given by the Northumbria Health care National Health Service Foundation Trust (C1792). The DISC Study was registered at clinicaltrials.gov as NCT01214681.

### Intervention protocol

The DISC Study was a double-blind, randomized, placebo-controlled dietary intervention in which 75 healthy participants were supplemented with RS and/or polydextrose for 50 d in a 2 × 2 factorial design. We aimed to test the impact of these NDCs on a panel of both novel and well-established biomarkers of colorectal health. The study was not subject to a formal power calculation, and a target of 75 participants, allowing for a 10% dropout rate, was set. This target of 75 participants was based on a previous study that detected significant effects on colonic crypt cell kinetics and gene expression after supplementing 65 CRC patients with RS for up to 4 wk (20). Although 15% of the randomly assigned participants were lost between assignment and the analysis stage, the target of 75 participants was met, with similar numbers of participants in each intervention group, reducing the potential for bias. Furthermore, there were no significant differences in baseline characteristics between the 75 analyzed participants and the 13 participants lost to follow-up (**Supplemental Table 1**).

At least 1 wk postendoscopy, participants were randomly assigned to 1 of 4 dietary interventions as follows:

- Double-placebo group: 12 g maltodextrin (DuPont Danisco) and 23 g Amioca starch (Ingredion, formerly National Starch, Food Innovation)
- RS group: 23 g Hi-maize 260 (Ingredion) and 12 g maltodextrin
- Polydextrose: 12 g Litesse *Ultra* (DuPont Danisco) and 23 g Amioca starch
- Double-intervention group (RS + polydextrose): 23 g Hi-maize 260 and 12 g Litesse *Ultra*

Participants were randomly assigned by selecting a sealed, opaque envelope labeled A, B, C, or D, and randomization was stratified by a preintervention endoscopy procedure to avoid confounding by different bowel preparations. The allocation codes were locked, and blinding was maintained until data collection and analyses were complete.

The intervention agents were provided in foil sachets that were labeled with a code and contained the agents in powder form. The participants were asked to consume 35 g intervention supplement/d, divided into 4 sachets (2 sachets of each agent). These were given in boxes containing a week’s worth of sachets. The supplements were added to cold foods or liquids, such as yogurt or orange juice, or mixed with cold water. Participants were asked to keep all of their sachets, both those that were consumed and those that were not, for assessment of compliance at the end of the intervention.

Preintervention rectal mucosal biopsies were collected at endoscopy (colonoscopy or flexible sigmoidoscopy) from the midrectum (10 cm from the anorectal verge). Postintervention rectal mucosal biopsies were collected by using rigid sigmoidoscopy from a similar location. Stool samples were collected 1 d before starting the dietary intervention and again on the last day of the intervention period. Samples were archived at −80°C within 18 h of collection. Anthropometric measurements (weight, height, waist circumference, and hip circumference) were taken at baseline and postintervention. Participants also completed a food-frequency questionnaire and lifestyle questionnaire that included information on physical activity and smoking status. The study remained blinded until all statistical analyses were complete.

### Preparation of fecal extracts and measurement of fecal calprotectin concentration

Stool samples were defrosted overnight and mixed thoroughly by using a Stomacher80 Biomaster (Seward Ltd). Fecal extracts were made with the use of a Fecal Sample Preparation Kit (Calpro AS) by using 100 mg stool according to the manufacturer’s instructions.

Fecal calprotectin was quantified by using the CALPROLAB Calprotectin ELISA (ALP) kit (Calpro AS) according to the manufacturer’s instructions by using fecal extracts diluted 1:20 in sample dilution buffer. Paired extracts (before and after intervention) were assayed on the sample plate. Optical density was read after 40 min incubation with enzyme substrate solution on a FLUOstar Omega microplate reader (BMG Labtech Ltd) operated by BMG Omega software version 1.20.

### Isolation of total RNA from rectal mucosal biopsies

Total RNA for mRNA analyses was extracted from half of a rectal mucosal biopsy by using the RNeasy Mini Kit (Qiagen) as described by the manufacturer. RNA including microRNA was extracted separately from half of a rectal mucosal biopsy by using the miRNeasy Mini Kit (Qiagen). Tissue disruption was performed by shaking the tissue samples with five 3-mm glass beads (VWR) for 1 min in Buffer RLT (RNeasy Mini Kit) or QIAzol Lysis Reagent (miRNeasy Mini Kit) by using an amalgamator. The lysate and beads were transferred to QiaShredders (Qiagen) for homogenization. RNA concentration and an indication of RNA purity were determined by using the NanoDrop 1000 spectrophotometer (Thermo Scientific) and NanoDrop 1000 Software version 3.7.1. RNA integrity was assessed by agarose gel electrophoresis.

### Quantification of Wnt pathway-related mRNA expression by qPCR

Two-step qRT-PCR was performed. cDNA was synthesized from 1 μg RNA by using the QuantiTect Reverse Transcription Kit (Qiagen) as described by the manufacturer. cDNA was diluted 10 times by using nuclease-free water to yield a total volume of 200 μL.

Gene expression was quantified by qPCR with the use of the Applied Biosystems StepOnePlus system. Expression of cyclin D1 (*CCND1*), v-myc avian myelocytomatosis viral oncogene homolog (*c-MYC*), *SFRP1*, and 2 reference genes, *18S* and *β2M*, was quantified as follows. Each reaction contained 5 μL ImmoMix (2×) (Bioline), 1 μL BSA (Ambion), 0.6 μL RNase-free water, 0.2 μL ROX (50×) (Invitrogen), 0.1 μL magnesium chloride (Bioline), 0.06 μL SYBR Green (100×) (Invitrogen), and 0.02 μL each forward and reverse primer (100 μM), to which 3 μL of the sample cDNA was added. PCR cycling was performed with a 10-min initial activation step at 95°C followed by 40 cycles of 30-s denaturation at 95°C, 30-s annealing at 60°C, 30-s extension at 72°C, and a final extension for 3 min at 72°C. Samples were analyzed in duplicate. Measures of adenomatous polyposis coli (*APC*), axis inhibition protein 2 (*AXIN2*), catenin β 1/β-catenin (*CTNNB1*), fos like 1 (*FOSL1*), glycogen synthase kinase 3 β (*GSK3β*), jun proto-oncogene (*c-JUN*),* SFRP2*, Wnt family member 5a (*Wnt5A*), Wnt family member 11 (*Wnt11*),* 18S*, and *β2M* abundance were obtained by using the QuantiTect SYBR Green PCR Kit (Qiagen) and QuantiTect primer assays as described in the manufacturer’s instructions. Each reaction contained 5 μL cDNA and 15 μL master mix.

### Quantification of microRNA expression by RT-PCR

Expression of the 10 selected microRNAs was performed by 2-step qRT-PCR. First, 0.8 μg RNA was reverse transcribed by using the miScript II RT Kit (Qiagen) and miScript HiSpec Buffer as described by the manufacturer. The synthesized cDNA was then diluted 10 times with nuclease-free water to yield a total volume of 200 μL.

MicroRNA expression and expression of 2 reference RNAs, *SNORD68* and *RNU-6*, were quantified by qPCR by using the Applied Biosystems StepOnePlus system and miScript SYBR Green PCR Kit (Qiagen) as described by the manufacturers.

### Isolation of DNA and bisulfite modification

DNA was extracted from half of a rectal mucosal biopsy by using a phenol-chloroform method. DNA purity and concentration were assessed by using the NanoDrop 1000 spectrophotometer and NanoDrop 1000 Software version 3.7.1. Bisulfite modification of DNA was performed by using the EZ DNA Methylation Gold Kit (Zymo Research) as described by the manufacturer.

### Quantification of *SFRP1* methylation by pyrosequencing

Bisulfite-modified DNA was amplified by PCR by using the PyroMark CpG assay primer (PM00035364) and HotStarTaq Master Mix (Qiagen) in a 24-μL total reaction volume. Pyrosequencing was performed in duplicate by using 10 μL PCR product and Pyromark Q96 reagents and run on the Pyromark Q96 ID pyrosequencer (Qiagen) as described by the manufacturer. Appropriate negative and positive controls, including unmethylated and 100% methylated DNA, were included.

### Statistical analyses

Postintervention differences in the analyzed outcomes between the intervention groups were investigated by using the ANOVA general linear model. Orthogonal contrasts allowed for the examination of the effects of each dietary agent (RS or polydextrose) individually and for a potential interaction effect (interaction between RS and polydextrose). When there was no significant interaction, the main effects are reported. The preintervention measurement was included as a covariate to adjust for any differences at baseline and age, sex, endoscopy procedure, BMI (in kg/m^2^), and smoking status were also included as covariates. In addition, when data were not normally distributed, postintervention microRNA expression data were analyzed as described above after using Box-Cox transformation and using the Kruskal-Wallis nonparametric test. These 2 approaches produced similar conclusions and for the sake of clarity the results of the Kruskal-Wallis analysis are reported, and data are presented as medians. The relation between age and *SFRP1* methylation was explored by using the Spearman rank correlation analysis.

## RESULTS

### Characteristics of study subjects

**FIGURE 1:**
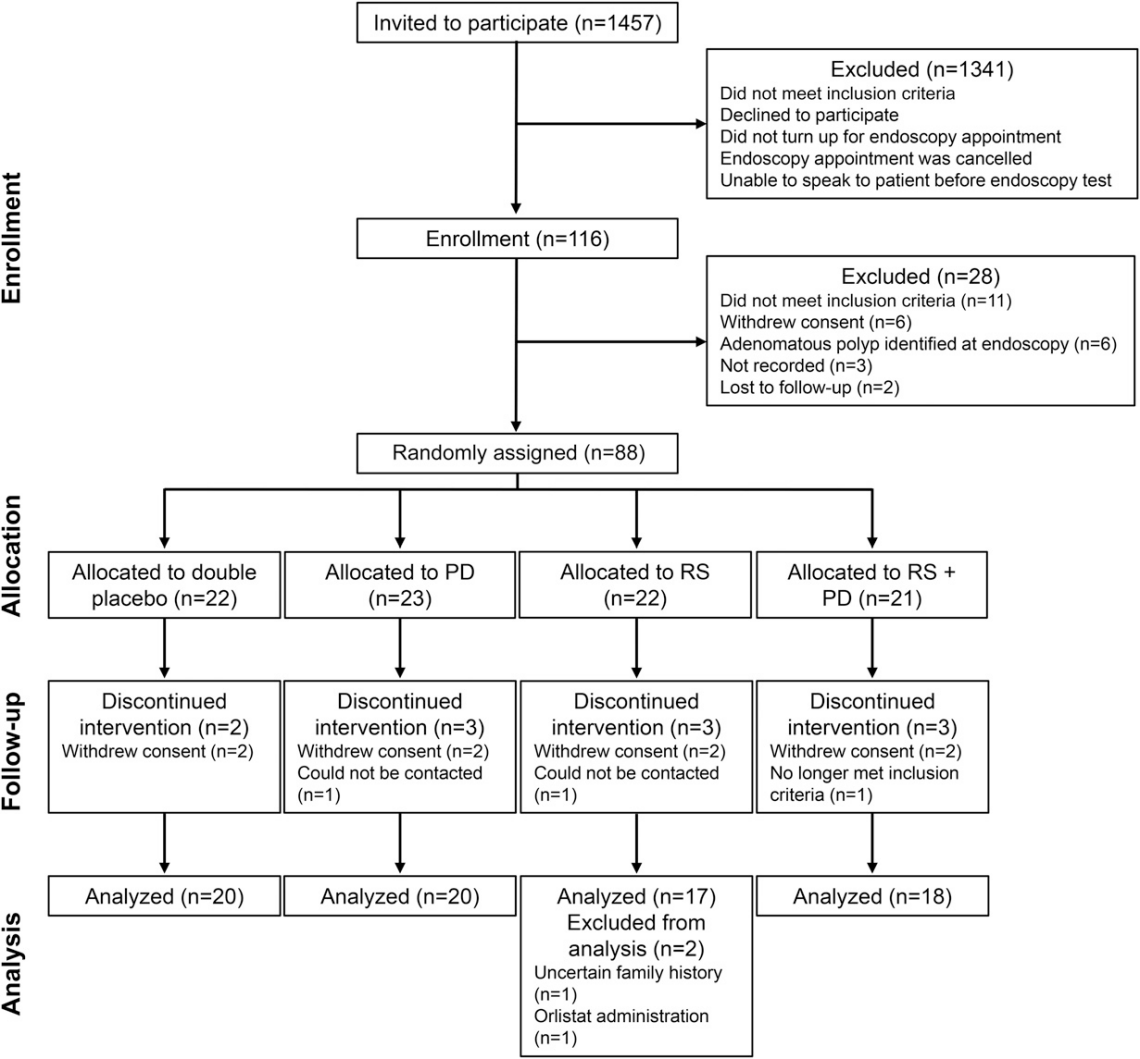
Consolidated Standards of Reporting Trials Diagram: flow of participants through the Dietary Intervention, Stem cells and Colorectal Cancer Study randomized controlled trial. PD, polydextrose; RS, resistant starch.

Eighty-eight healthy participants were recruited and randomly assigned to the intervention. The flow of participants throughout the DISC Study is shown in the Consolidated Standards of Reporting Trials diagram (**Figure 1**). Following random assignment, 11 participants (12.5%), distributed equally among the intervention groups, were lost to follow-up. At the end of the study, 2 participants from the RS group were excluded at the analysis stage because one had an uncertain family history of CRC and the other was taking orlistat, an inhibitor of fat digestion. In total, data for 75 participants were analyzed, representing a loss of 15% of participants between assignment and data analysis. As shown in Supplemental Table 1, there were no significant differences in baseline participant characteristics between the 13 participants lost to follow-up and the 75 analyzed participants, except for endoscopy procedure. All of the 13 participants lost to follow-up underwent endoscopic examination by flexible sigmoidoscopy, whereas for the 75 participants analyzed, 69% had flexible sigmoidoscopies and 31% had colonoscopies.

The number of participants in each intervention group was similar (20 in the double-placebo group, 20 in the polydextrose group, 17 in the RS group, and 18 in the RS + polydextrose group) (**Table 1**). The mean participant age across all 4 groups was 52.4 y, with the youngest and oldest participants aged 30 and 80 y, respectively. A large proportion of participants were overweight (36%) or obese (47%), and only 17% of the participants had a BMI within the normal range. There were no effects of RS or polydextrose on participant body weight (**Supplemental Table 2**). To minimize any confounding effects of intervention group differences in age, sex, endoscopy procedure, BMI, or smoking status on the measured outcomes, these variables were included as covariates during statistical analyses.

**TABLE 1:** Baseline characteristics of participants in the DISC Study randomly assigned to the 4 intervention groups^1^

	All (*n* = 75)	Double placebo (*n* = 20)	PD + RS placebo (*n* = 20)	RS + PD placebo (*n* = 17)	RS + PD (*n* = 18)
Ethnicity					
Caucasian	73 (97)^2^	19 (95)	19 (95)	17 (100)	18 (100)
Black African	1 (1.33)	1 (5)	0 (0)	0 (0)	0 (0)
Mixed race	1 (1.33)	0 (0)	1 (5)	0 (0)	0 (0)
Sex					
Female	40 (53)	10 (50)	11 (55)	13 (76)	6 (33)
Male	35 (47)	10 (50)	9 (45)	4 (24)	12 (67)
Age, y	52.4 (30–80)^3^	48.0 (30–70)	58.3 (33–80)	53.4 (42–67)	50.1 (36–74)
Smoking status					
Never	38 (51)	12 (60)	12 (60)	8 (47)	6 (33)
Former	21 (28)	4 (20)	5 (25)	6 (35)	6 (33)
Current	16 (21)	4 (20)	3 (15)	3 (18)	6 (33)
BMI, kg/m^2^	30.0 (23.0–49.3)	29.8 (23.2–37.1)	29.7 (23.2–49.3)	30.9 (23.0–43.1)	29.9 (23.4–41.8)
Procedure					
Colonoscopy	23 (31)	6 (30)	6 (30)	6 (35)	5 (28)
Flexible sigmoidoscopy	52 (69)	14 (70)	14 (70)	11 (65)	13 (72)

1 DISC, Dietary Intervention, Stem cells and Colorectal Cancer; PD, polydextrose; RS, resistant starch.

2 *n*; percentage in parentheses (all such values).

3 Mean; range in parentheses (all such values).

### Effects of RS and polydextrose on fecal calprotectin

Fecal calprotectin, a marker of local inflammation, was used as the primary outcome for this intervention study. Pre- and postintervention fecal calprotectin concentrations were determined by ELISA. There were no significant differences in fecal calprotectin between the intervention groups at baseline (*P* = 0.914). The effects of the interventions were analyzed by using the ANOVA general linear model, with preintervention calprotectin concentration included as a covariate. There were no significant effects of RS or polydextrose or an interaction between the 2 intervention agents on fecal calprotectin concentration (**Table 2**).

**TABLE 2:** Effects of supplementation with RS and PD on fecal calprotectin concentration^1^

	Effect of RS			Effect of PD		
	−	+	*P*	−	+	*P*
Fecal calprotectin, mg/kg	35.8 ± 7.7	49.3 ± 8.5	0.249	33.0 ± 8.3	52.0 ± 8.11	0.114

1 Values are least-squares means ± SEMs (*n* = 75) for the effect of the absence (−) or presence (+) of the specified nondigestible carbohydrate on fecal calprotectin concentrations. *P* values calculated by the ANOVA general linear model. PD, polydextrose; RS, resistant starch.

### Effects of RS and polydextrose on the expression of Wnt pathway-related genes

**FIGURE 2:**
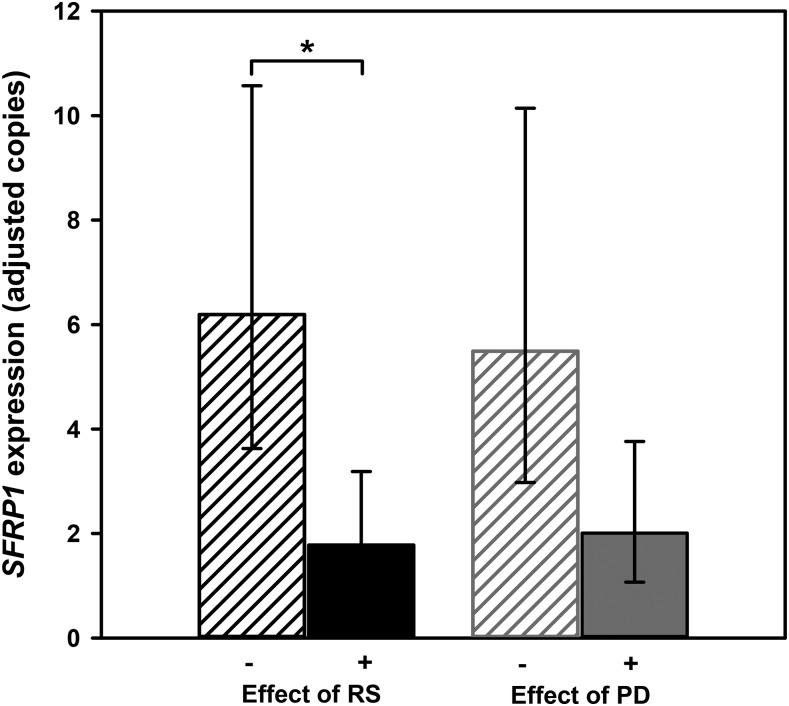
Effects of the absence (−) or presence (+) of the specified nondigestible carbohydrate on *SFRP1* mRNA expression in the human rectal mucosa. Data are least-squares means of *SFRP1* expression postintervention after adjusting for covariates, and error bars represent 95% CIs. *P < 0.05, significant effect of agent (ANOVA general linear model) (*n* = 39). There were no significant interactions. PD, polydextrose; RS, resistant starch; *SFRP1*, secreted frizzled-related protein 1.

To determine the effects of the dietary intervention on the Wnt signaling pathway, expression of a panel of 12 Wnt pathway-related genes was quantified pre- and postintervention in rectal mucosal biopsies by using qRT-PCR (**Table 3**). RS significantly reduced the expression of *CTNNB1*, encoding β-catenin, by 25% (*P* = 0.045). Participants supplemented with RS also had significantly lower *c-MYC* expression (*P* = 0.037), and polydextrose supplementation reduced *SFRP2* expression (*P* = 0.01). Supplementation with RS and with polydextrose reduced *SFRP1* expression by 71% (*P* = 0.005) and 63% (*P* = 0.053), respectively (**Figure 2**).

**TABLE 3:** Effects of supplementation with RS and PD on Wnt pathway–related gene expression in the human rectal mucosa^1^

	Effect of RS			Effect of PD		
Gene	−	+	*P*	−	+	*P*
*APC*	1.83 (1.48, 2.22)	1.49 (1.17, 1.85)	0.227	1.55 (1.24, 1.89)	1.77 (1.43, 2.17)	0.410
*AXIN2*	2.15 (1.81, 2.50)	2.22 (1.89, 2.55)	0.230	2.31 (1.98, 2.65)	2.42 (2.07, 2.77)	0.682
*CCND1*	66.5 (45.9, 96.3)	45.2 (30.6, 66.7)	0.171	50.9 (33.4, 77.6)	59.0 (37.7, 92.4)	0.678
*CTNNB1*	14.9 (12.3, 17.7)	11.1 (9.0, 13.5)	0.045	12.5 (10.3, 15.1)	13.3 (10.8, 16.0)	0.679
*FOSL1*	0.023 (0.01, 0.10)	0.01 (0.01, 0.03)	0.474	0.01 (0.00, 0.02)	0.03 (0.01, 0.17)	0.303
*GSK3β*	5.31 (4.56, 6.07)	4.08 (4.08, 5.53)	0.351	4.92 (4.18, 5.65)	5.20 (4.44, 5.96)	0.615
*c-JUN*	8.20 (6.54, 10.04)	7.23 (5.73, 8.91)	0.436	6.96 (5.48, 8.63)	8.48 (6.78, 10.37)	0.230
*c-MYC*	10.18 (6.97, 14.85)	5.41 (3.58, 8.19)	0.037	8.06 (5.01, 12.59)	6.84 (4.37, 10.73)	0.668
*SFRP1*	6.19 (3.36, 10.57)	1.78 (1.00, 3.18)	0.005	5.49 (2.97, 10.14)	2.01 (1.07, 3.76)	0.053
*SFRP2*	0.007 (0.004, 0.012)	0.007 (0.004, 0.012)	0.980	0.012 (0.007, 0.021)	0.004 (0.002, 0.007)	0.010
*Wnt5A*	0.13 (0.01, 0.18)	0.10 (0.08, 0.01)	0.267	0.10 (0.08, 0.14)	0.13 (0.10, 0.18)	0.332
*Wnt11*	0.12 (0.09, 0.15)	0.09 (0.07, 0.11)	0.145	0.10 (0.08, 0.13)	0.10 (0.08, 0.13)	0.910

1 Values are least-squares means (95% CIs) for adjusted values (2^−ΔCt^ × 10,000) relative to the *18S* and *β2M* reference genes for the effect of the absence (−) or presence (+) of the specified nondigestible carbohydrate on expression of *APC* (*n* = 35), *AXIN2* (*n* = 66), *CCND1* (*n* = 35), *CTNNB1* (*n* = 63), *FOSL1* (*n* = 16), *GSK3β* (*n* = 64), *c-JUN* (*n* = 64), *c-MYC* (*n* = 37), *SFRP1* (*n* = 39), *SFRP2* (*n* = 49), *Wnt5A* (*n* = 63), and *Wnt11* (*n* = 42). *P* values calculated by the ANOVA general linear model. *APC*, adenomatous polyposis coli; *AXIN2*, axis inhibition protein 2; *CCND1*, cyclin D1; *c-JUN*, jun proto-oncogene; *c-MYC*, v-myc avian myelocytomatosis viral oncogene homolog; *CTNNB1*, catenin β 1/β-catenin; *FOSL1*, fos like 1; *GSK3β*, glycogen synthase kinase 3 β PD, polydextrose; RS, resistant starch; *SFRP1*, secreted frizzled-related protein 1; *SFRP2*, secreted frizzled-related protein 2; *Wnt5A*, Wnt family member 5a; *Wnt11*, Wnt family member 11.

### Effects of RS and polydextrose on *SFRP1* methylation

As both RS and polydextrose modulated *SFRP1* expression, we investigated whether these effects were mediated via epigenetic mechanisms. We hypothesized that downregulation of *SFRP1* mRNA expression resulted from increased DNA methylation of the *SFRP1* promoter, causing transcriptional silencing, and tested this hypothesis by quantifying methylation at 7 CpG sites within the *SFRP1* promoter by pyrosequencing.

**FIGURE 3:**
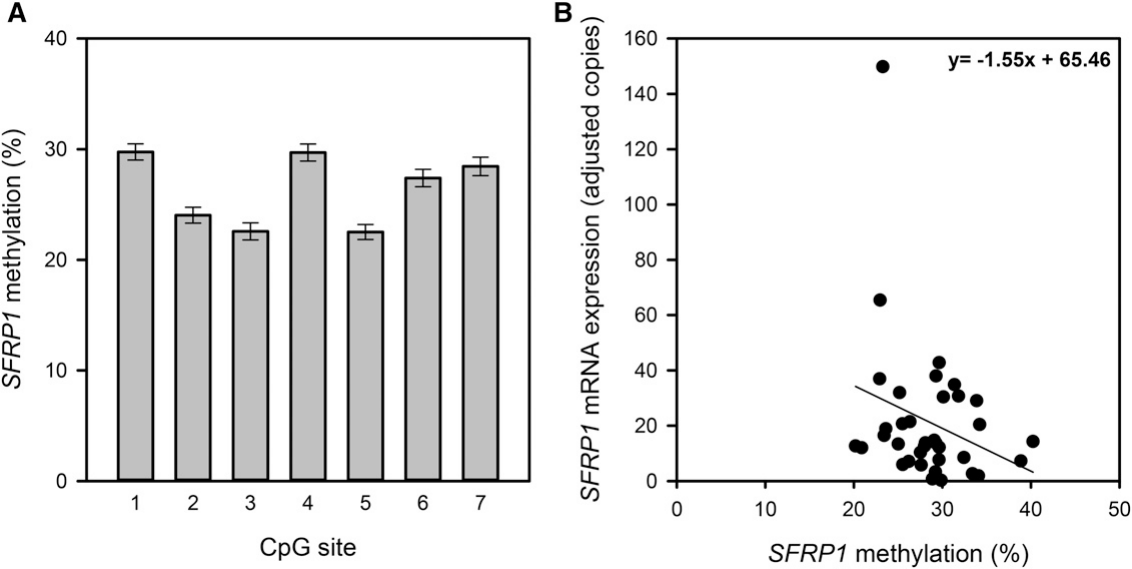
*SFRP1* methylation (A) at each of the 7 CpG sites quantified within the *SFRP1* promoter region in DNA from rectal mucosal biopsies for participants at baseline (*n* = 69). Error bars represent SEMs. (B) Inverse relation between the mean methylation of 7 CpG sites within the *SFRP1* promoter and *SFRP1* mRNA expression in the human rectal mucosa at baseline (Spearman rank correlation analysis) (*n* = 36; *R*^2^ = 0.073; *P* = 0.240). *SFRP1*, secreted frizzled-related protein 1.

At baseline, *SFRP1* promoter methylation was similar for all treatment groups with an overall mean methylation across all 7 CpG sites of 26.3%. In addition, baseline methylation varied relatively little across CpG sites with the lowest at CpG site 5 (22.5%) and highest at CpG site 1 (29.8%) (**Figure 3A**). At baseline, there was no convincing evidence for a correlation (*R*^2^ = 0.073; *P* = 0.240) between *SFRP1* methylation and *SFRP1* mRNA expression (Figure 3B).

Supplementation with RS and polydextrose for 50 d did not affect *SFRP1* methylation at any of the 7 CpG sites (**Supplemental Table 3**). However, when averaged across all CpG sites, there was a trend for a reduction in *SFRP1* methylation with polydextrose supplementation by 2.5% (*P* = 0.055). Interestingly, postintervention *SFRP1* methylation and mRNA expression tended to be positively correlated (*R*^2^ = 0.01; *P* = 0.067, data not shown).

### Selection of microRNAs that target *SFRP1* for quantification by qRT-PCR

**FIGURE 4:**
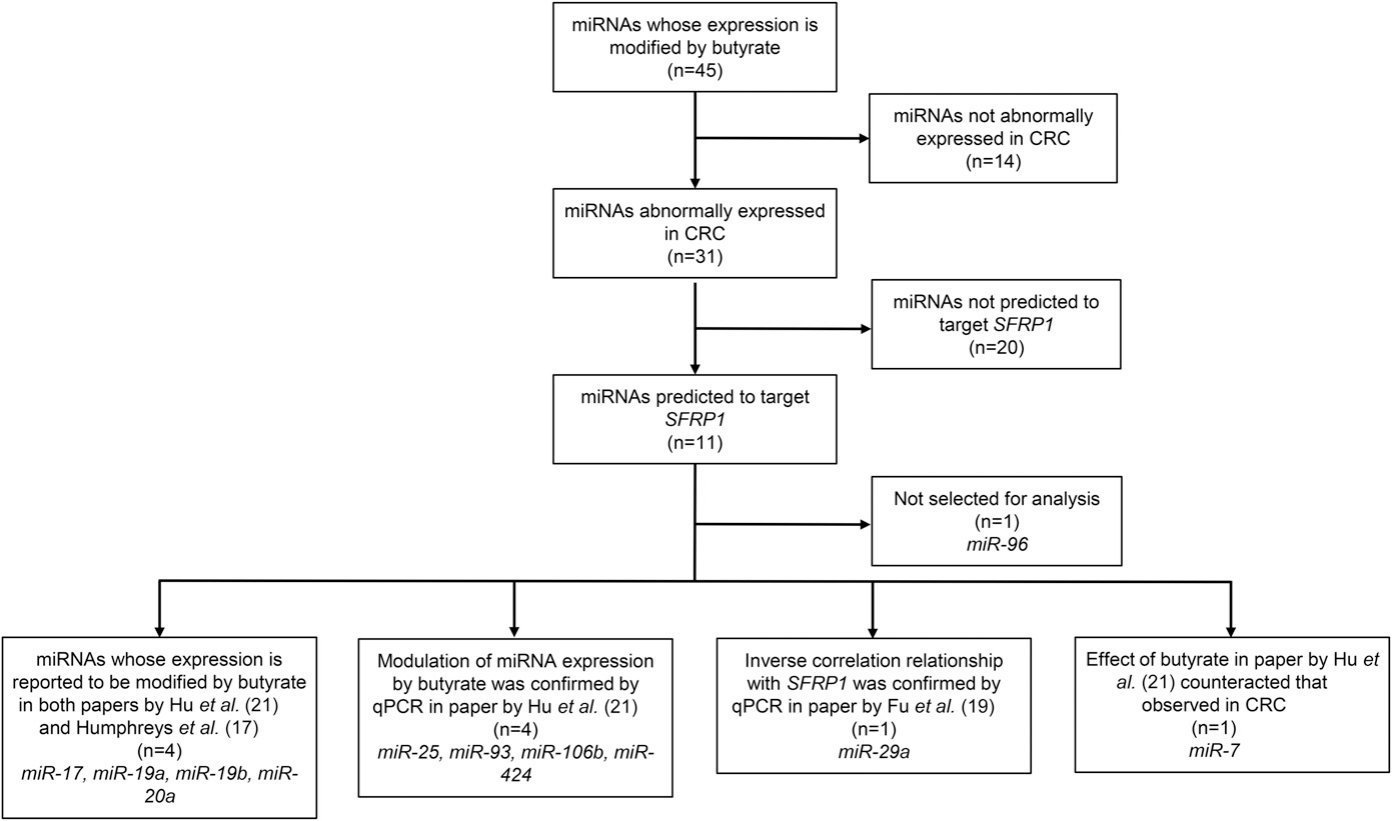
Flowchart illustrating the literature-based process used to select the 10 target miRNAs. Data are from Humphreys et al. (17), Fu et al. (19), and Hu et al. (21). CRC, colorectal cancer; miRNA, microRNA; qPCR, quantitative polymerase chain reaction; *SFRP1*, secreted frizzled-related protein 1.

A multistep approach was conducted to select 10 microRNAs predicted to target *SFRP1*. First, we searched the literature for studies that investigated effects of butyrate (a likely mediator of effects of NDCs) on microRNA expression in the colorectal mucosa and identified 2 studies (17, 21) reporting a total of 45 microRNAs that were modulated by butyrate. Second, we searched the literature for evidence that any of these 45 microRNAs modulated by butyrate were abnormally expressed in CRC and found that 31 were reported to be abnormally expressed in CRCs. Third, we searched the literature for evidence of microRNAs that may regulate *SFRP1* expression in the large bowel and identified one article that reported 16 microRNAs that were inversely correlated with *SFRP1* mRNA expression (19). From the 31 microRNAs that were both modified by butyrate and abnormally expressed in CRCs, 11 showed a significant inverse correlation with *SFRP1* and therefore were predicted to target *SFRP1.* From these 11 microRNAs, 10 were selected to be quantified by qRT-PCR (**Figure 4**).

### Effects of RS and polydextrose on expression of microRNAs predicted to target *SFRP1*

**FIGURE 5:**
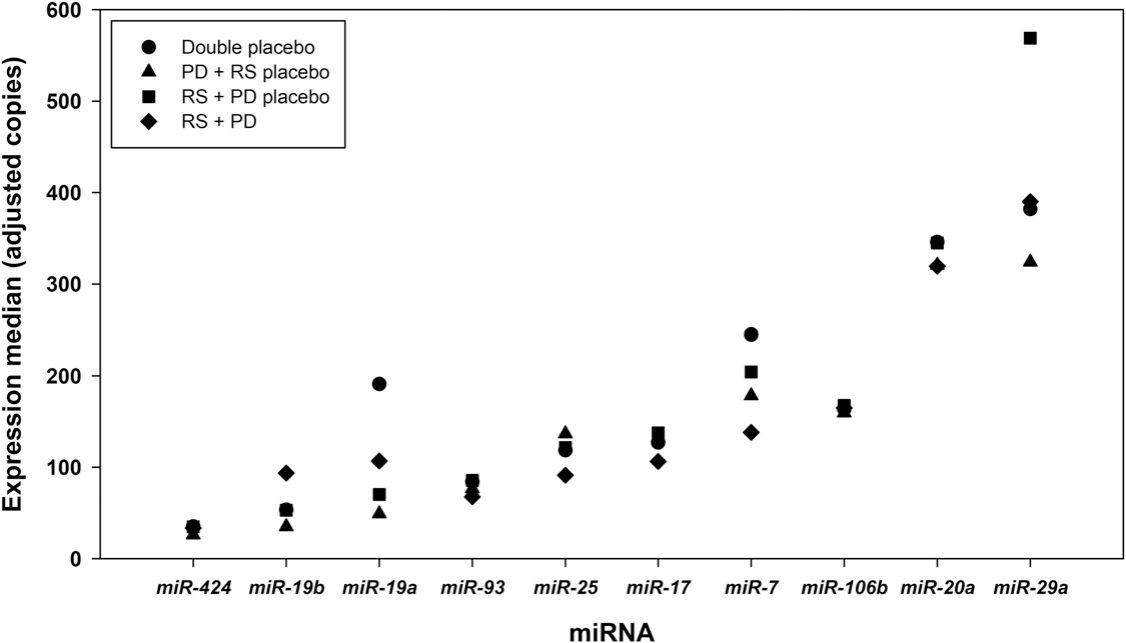
Expression of 10 selected miRNAs in the human rectal mucosa at baseline for each of the 4 intervention groups. Data are presented as medians (*n* = 53). miRNA, microRNA; PD, polydextrose; RS, resistant starch.

Ten microRNAs previously reported to be abnormally expressed in CRC, for which expression is altered by butyrate treatment and that have been predicted to target *SFRP1,* were quantified by qPCR. At baseline, colorectal mucosal expression was lowest for *miR-424* and highest for *miR-29a* but did not differ significantly between the 4 intervention groups (**Figure 5**).

Effects of RS and polydextrose on microRNA expression were analyzed by using the Kruskal-Wallis nonparametric test. There was no evidence that RS or polydextrose supplementation affected the expression of any of the 10 microRNAs (**Supplemental Table 4**).

### Relation between age and *SFRP1* promoter methylation

**FIGURE 6:**
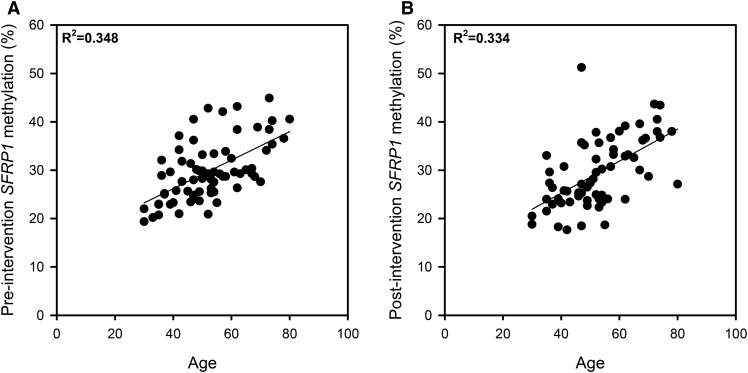
Positive correlations between age and mean *SFRP1* methylation across all 7 CpG sites (A) preintervention (*n* = 70; *P* < 0.001) and (B) postintervention (*n* = 65; *P* < 0.01) (Spearman rank correlation analysis). *SFRP1*, secreted frizzled-related protein 1.

CRC is an age-related disease, and age-related changes in methylation are associated with carcinogenesis (22). Previous studies have reported increased *SFRP1* methylation with age in both normal and tumor tissues (23, 24). Using data from the present study, we observed a significant positive relation between age and *SFRP1* methylation at all CpG sites for both pre- and postintervention data. The positive correlations between age and mean *SFRP1* methylation across all 7 CpG sites for preintervention (*R*^2^ = 0.348; *ρ*= 0.565; *P* < 0.001) and postintervention (*R*^2^ = 0.334; *ρ*= 0.592; *P* < 0.001) data are illustrated in **Figure 6**. At baseline, there was also a trend for an inverse relation between age and *SFRP1* mRNA expression (*ρ* = −0.294; *P* < 0.069, data not shown).

## DISCUSSION

We report findings from the first randomized, double-blind, placebo-controlled intervention study (the DISC Study) investigating the effects of supplementation with 2 NDCs, RS and polydextrose, on fecal calprotectin concentration, Wnt pathway-related gene expression, and the epigenetic regulation of *SFRP1* in the rectal mucosa of healthy individuals. We hypothesized that NDCs may have protective effects in the large bowel by reducing inflammation, but we did not observe any effects of RS and/or polydextrose on concentrations of fecal calprotectin, an indicator of inflammation in the large bowel. Evidence in the literature is mixed. One study reported an inverse correlation between fiber intake and fecal calprotectin concentration (25), whereas another reported an increase in calprotectin with RS supplementation (26). In this context, fecal calprotectin may be an imperfect marker of mucosal inflammation, and direct measures of other inflammatory markers in mucosal biopsies could be more informative.

RS supplementation downregulated expression of β-catenin, a key player in the canonical pathway, and the target gene *c-MYC*, suggesting a reduction in Wnt pathway activity. In addition, supplementation with RS and polydextrose reduced *SFRP1* expression by 70% and 63%, respectively, and polydextrose, but not RS, reduced *SFRP2* expression. As *SFRP1* is a negative regulator of Wnt signaling, reduced mRNA expression, if paralleled at the protein level, would alleviate Wnt pathway inhibition and consequently increase Wnt signaling. Because hyperactive Wnt signaling is associated with colorectal carcinogenesis (27), our observation that RS and polydextrose suppressed *SFRP1* expression is counterintuitive because higher consumption of NDCs is associated with lower CRC risk (4). However, not all types of NDCs may protect against CRC (28), and studies that have investigated the effect of NDCs on indicators of large-bowel health and CRC risk have yielded inconsistent findings (29–35).

In patients with CRC, supplementation with RS for up to 4 wk reduced the proportion of mitotic cells in the upper half of colonic crypts, a marker of crypt health, and positively influenced the expression of 2 cell cycle regulators, CDK4 and GADD45A, providing evidence for antineoplastic effects of RS (20). Similar antineoplastic effects have been observed with polydextrose in some studies. Treatment of Caco-2 adenocarcinoma cells with polydextrose fermentation products induced apoptosis and reduced proliferation (36), and rats fed a polydextrose-containing diet had a significantly reduced aberrant crypt foci (ACF) formation in the colorectum (31). In contrast, other studies have shown no effect of NDC supplementation on CRC risk. For example, the CAPP (Colorectal Adenoma/Carcinoma Prevention Programme) 1 Study showed that supplementation with RS for 17 mo did not affect polyp number or size in young patients with familial adenomatous polyposis (30). In the CAPP2 Study, patients with Lynch Syndrome were supplemented with RS for up to 4 y, and no effects on CRC or on other Lynch Syndrome cancers were observed (29). Similarly, studies have shown no protective effects of polydextrose against the formation of ACF or tumors in carcinogen-treated rats and mice, respectively (34, 35). Finally, some animal studies have suggested adverse effects of NDC supplementation. Feeding RS for 5 mo increased the number of intestinal tumors in *Apc*1638N mice (32), and feeding raw potato starch alone for 31 wk increased the number of ACF and tumors in rats (33). It is difficult to reconcile these conflicting findings, but the evidence suggests that the consequences for CRC development will depend on the nature, dose, and duration of supplementation with specific NDCs and on the species and genotype of the recipient.

The observation that *SFRP1* expression was reduced by RS and polydextrose is consistent with previous evidence that in healthy cells butyrate stimulates Wnt signaling, leading to regulation of multiple processes (7, 37). In contrast, in gastric cancer cells Shin and colleagues (13) demonstrated that butyrate induced *SFRP1*-promoter demethylation and restored expression levels. The differing effects of butyrate in healthy and cancer cells, referred to as the butyrate paradox, are well documented and suggest that the effects of this metabolite are highly cell-type specific (38). In the normal mucosa, butyrate appears to stimulate Wnt signaling through the reduction of *SFRP1* and other Wnt antagonists. Evidence for the effects of butyrate in neoplastic colorectal cells is conflicting. Butyrate has been shown to downregulate Wnt activity in CC531 rat colon carcinoma cells by reducing the expression of 4 Wnt target genes that are upregulated in CRC [*CCND1*,* c-MYC*,* FOSL1*, and follistatin (*FST*)] (39), but it has also been shown to induce Wnt signaling leading to stimulated apoptosis (40–42). Consequently, Bordonaro and colleagues (7) have proposed that there is a gradient of Wnt activity in the large bowel; inactive Wnt signaling is associated with stimulated cell differentiation and apoptosis, low levels of Wnt activity result in tissue renewal, moderate levels of Wnt activity lead to uncontrolled proliferation, and hyperactive Wnt signaling stimulates apoptosis. This proposal would explain why cells exhibiting hyperactivation of the Wnt pathway in response to butyrate treatment showed greater levels of apoptosis than those that did not show hyperactive Wnt signaling (40).

Epigenetic mechanisms encompass an integrated set of marks (DNA methylation and posttranslational modifications of histones) and molecules (including microRNAs) which regulate gene expression (43) and are responsive to dietary and other environmental factors (44). We hypothesized that the effects of RS and polydextrose on *SFRP1* expression were mediated via altered methylation of the *SFRP1* promoter and changes in abundance of microRNAs that target *SFRP1*. We attempted to test this hypothesis by quantifying DNA methylation at 7 CpG sites within the promoter region of *SFRP1*. At baseline, although not statistically significant, we observed inverse correlations between *SFRP1* methylation and mRNA expression at each of the 7 CpG sites, which is in accord with the observation that gene expression levels reduce with increasing DNA methylation. However, postintervention expression levels correlated positively with *SFRP1* methylation, suggesting that downregulation of *SFRP1* expression with RS and polydextrose resulted from mechanisms other than effects on DNA methylation. There were no significant effects of supplementation with RS or polydextrose on *SFRP1* methylation at any of the individual CpG sites or on the mean methylation across all sites. We observed that *SFRP1* promoter methylation increased significantly with participant age, which confirms findings in colorectal tissue from healthy and cancer patients (24, 45).

To investigate transcriptional regulation of *SFRP1* by microRNAs, we selected 10 microRNAs that met the criteria of being modulated by butyrate, abnormally expressed in CRC and predicted to target *SFRP1*. Because supplementation with RS and polydextrose reduced *SFRP1* expression, we hypothesized that such supplementation would increase expression of microRNAs that target *SFRP1*. We observed no significant effects of RS or polydextrose on the expression of selected microRNAs predicted to regulate *SFRP1.* Indeed, there was a trend toward reduced expression of *miR-17*,* miR-19a*,* miR-19b*, and *miR-20a* (members of the oncogenic *miR-17–92* cluster) with RS supplementation, which counters our hypothesis. However, because members of the *miR-17–92* cluster are frequently upregulated in CRC, modulation of oncogenic microRNA expression could be another possible mechanism through which butyrate and NDCs may protect against CRC. High intake of red meat, a dietary risk factor for CRC, increased oncogenic microRNA expression, whereas supplementation of a high–red meat diet with butyrylated high-amylose maize starch (RS type 2) restored microRNA levels to baseline levels, suggesting that RS reverses the detrimental effects associated with red meat (18). In the current study, RS and polydextrose may have reduced *SFRP1* expression via histone modifications because butyrate is a well-established histone deacetylase inhibitor (46), but a lack of sufficient tissue precluded our investigation of potential histone modifications.

In conclusion, supplementation with RS or polydextrose for 50 d had no effect on fecal calprotectin concentration. However, NDC supplementation reduced the expression of *SFRP1* but did not affect the expression of the other 11 quantified Wnt pathway-related genes. The observed effects of RS and polydextrose on *SFRP1* expression suggest the potential for reduced inhibition of Wnt activation, but the effects on Wnt pathway activity and subsequent physiologic effects, e.g., on crypt cell proliferation, remain to be investigated. Because fermentation of NDCs, such as RS and polydextrose, by the large-bowel microbiota yields the epigenetic regulator butyrate as an end product, we hypothesized that the observed transcriptional changes in *SFRP1* would be mediated by epigenetic mechanisms. We found no evidence that RS or polydextrose altered methylation of the *SFRP1* promoter, but our results did not exclude the possibility that the NDCs altered methylation at other sites, e.g., at intragenic regions and at CpG island shores (>2 kb upstream of the promoter region), which have been linked with changes in gene expression (47, 48). The DISC Study was not subject to a formal power calculation, so it is possible that our study was underpowered to detect effects of RS and polydextrose on some of the measured outcomes. Furthermore, although our dietary intervention did not affect expression of the selected microRNAs, there may have been intervention effects on microRNAs not investigated here. The deciphering of microRNAs that target specific genes is complex because a single gene may be regulated by a large number of microRNAs and, likewise, a single microRNA can have numerous targets.

## Footnotes

7 Abbreviations used: ACF, aberrant crypt foci; *c-MYC*, v-myc avian myelocytomatosis viral oncogene homolog; CRC, colorectal cancer; DISC, Dietary Intervention, Stem cells and Colorectal Cancer; NDC, nondigestible carbohydrate; RS, resistant starch; SFRP, secreted frizzled-related protein.
